# The Correlation Between Apathy and the Efficacy of Rehabilitation in Patients With Parkinson's Disease: A Retrospective Observational Study

**DOI:** 10.1002/brb3.71480

**Published:** 2026-05-14

**Authors:** Jinping Fang, Keke Chen, Yonghong Liu, Ruidan Wang, Yixuan Wang, Detao Meng, Hongyu Zhang, Tingting Hou, Hongjiao Yan, Xia An, Yi Zhen, Zhenzhen Li, Cuiping Xue, Boyan Fang

**Affiliations:** ^1^ Parkinson Medical Center, Beijing Rehabilitation Hospital Capital Medical University Beijing China; ^2^ Beijing Rehabilitation Medical College Capital Medical University Beijing China; ^3^ Department of Rehabilitation, Beijing Rehabilitation Hospital Capital Medical University Beijing China

**Keywords:** apathy, mechanisms, Parkinson's disease, rehabilitation

## Abstract

**Background:**

Apathy impacts mood and social functioning in patients with Parkinson's disease (PWP), and its effect on motor rehabilitation outcomes remains unexplored.

**Objective:**

This study evaluates whether the presence of apathy (apathy status) is associated with rehabilitation outcomes in PWP.

**Methods:**

We retrospectively analyzed records of 122 PWP (Hoehn–Yahr stage ≤ 3) who underwent a 2‐week multidisciplinary intensive rehabilitation treatment (MIRT) between April 2020 and July 2024, dividing them into Parkinson's disease with apathy (PDA+, score > 14) and without apathy (PDA−, score ≤ 14) based on Modified Apathy Evaluation Scale (MAES). The outcome measures included the Movement Disorder Society Unified Parkinson's Disease Rating Scale part III (MDS‐UPDRS III), the Modified Parkinson Activity Scale (M‐PAS), the Five Times Sit to Stand Test (FTSTS), the Timed Up and Go Test (TUG), and the 10‐Meter Walk Test (10MWT). We conducted group comparisons and employed multiple linear regression analyses, treating the MAES both as a dichotomous variable and as a continuous predictor. In these analyses, we adjusted for baseline measures of MDS‐UPDRS III, FTSTS, and TUG. Additionally, we performed sensitivity analyses using alternative cutoffs (MAES > 13 and > 15).

**Results:**

Following MIRT, the PDA– group (*n* = 75) showed significant improvements in MDS‐UPDRS III, M‐PAS, FTSTS, and TUG (*p* < 0.001, *p *< 0.001, *p* = 0.011, and *p* < 0.001, respectively). In contrast, no such improvements were observed in the PDA+ group (*n* = 47). The improvements in MDS‐UPDRS III, FTSTS, and TUG scores exhibited significant differences between the groups (*p* = 0.018, *p* = 0.046, and *p* = 0.042, respectively). In multivariable linear regression with MAES as a continuous variable, higher MAES was independently associated with smaller ΔMDS‐UPDRS III (*β* = 0.147, 95% CI 0.017–0.277, *p* = 0.027; *R* ^2^ = 0.145), after adjustment for baseline MDS‐UPDRS III, FTSTS, and TUG. Modeling MAES dichotomously (> 14 vs. ≤ 14) produced similar results (*β* = 2.378, 95% CI 0.341–4.415, *p* = 0.023; *R* ^2^ = 0.148). Sensitivity analyses using cutoffs of > 13 and > 15 yielded qualitatively comparable findings.

**Conclusions:**

Baseline apathy severity was associated with attenuated motor improvement after MIRT in PWP; understanding its mechanisms is crucial for personalized rehabilitation and treatment effectiveness.

**Trial Registration:**

ClinicalTrials.gov identifier: ChiCTR2000033768, 2020/06/11

## Graphical abstract

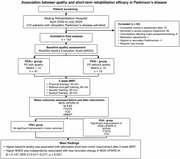

**Abbreviations**
10MWT: 10‐Meter Walk Test
FTSTS: Five Times Sit to Stand Test
HAMD: Hamilton Depression Scale
HARS: Hamilton Anxiety Rating Scale
H‐Y: Hoehn–Yahr
LEDD: levodopa equivalent daily doses
MAES: Modified Apathy Evaluation Scale
MDS‐UPDRS III: Movement Disorder Society Unified Parkinson's Disease Rating Scale part III
MIRT: multidisciplinary intensive rehabilitation treatment
MoCA: Montreal Cognitive Assessment
M‐PAS: Modified Parkinson Activity Scale
PD: Parkinson's disease
PFS⁃16: Parkinson Fatigue Scale
PWP: patients with Parkinson's disease
TUG: Timed Up and Go Test

## 1 Introduction

Parkinson's disease (PD) is a progressive neurodegenerative disorder characterized by motor symptoms such as tremor, rigidity, and bradykinesia. Recent research has underscored the substantial influence of non‐motor symptoms, including cognitive impairment, depression, and apathy, on patients' quality of life and disease prognosis (Dorsey and Bloem 2018; Bloem et al. 2021; Hinkle et al. 2021). Apathy, one of the core non‐motor symptoms of PD, is marked by diminished motivation, emotional responsiveness, and goal‐directed behavior, affecting between 17% and 70% of individuals diagnosed with PD (Levy 2012; Mele et al. 2020). Current evidence indicates that the pathological mechanism underlying apathy in PD is associated with dysfunction of the prefrontal‐basal ganglia circuits, predominantly due to dopaminergic neuronal damage (Pagonabarraga et al. 2015). Although rehabilitation interventions, such as physical therapy and exercise training, have demonstrated efficacy in improving both motor and non‐motor symptoms in individuals with PD (Bloem et al. 2015; Langeskov‐Christensen et al. 2024), individual differences in adherence to rehabilitation programs and engagement motivation can significantly impact treatment outcomes (Afshari et al. 2017). Given its significant impact on motivation and participation, apathy may be a critical factor influencing the effectiveness of rehabilitation interventions. Recent advances in neuropsychology have highlighted the role of motivational deficits in neurological rehabilitation (Saleh et al. 2021; Hoy et al. 2024). Specifically, studies focusing on post‐stroke rehabilitation have shown that patients exhibiting apathy experience markedly slower motor recovery, indicating that motivational states serve as a pivotal modulator of neurorehabilitation outcomes (Hama et al. 2007).

Multidisciplinary intensive rehabilitation treatment (MIRT) is a comprehensive, intensive rehabilitation program that integrates aerobic exercise with multidisciplinary interventions to address the specific needs of patients with Parkinson's disease (PWP) (Brötz 2013). A substantial body of research has demonstrated its significant short‐ and long‐term benefits for both motor and non‐motor symptoms of PD (Trend et al. 2002; Ferrazzoli et al. 2018; Chen et al. 2021). Despite the established benefits of rehabilitation exercises in improving PD symptoms, the impact of factors such as apathy on treatment outcomes remains poorly understood. This study aims to investigate the relationship between the presence of apathy and the effectiveness of rehabilitation exercises in PWP, particularly focusing on whether apathy influences motor function improvement during rehabilitation. By elucidating this relationship, we aim to provide clinicians with deeper insights to facilitate more effective adjustment of treatment plans.

## 2 Materials and Methods

This retrospective observational study was reported in accordance with the STROBE (Strengthening the Reporting of Observational Studies in Epidemiology) guidelines.

### 2.1 Participants

We retrospectively analyzed the medical records of the PWP who participated the cohort study, underwent MIRT at Neurological Rehabilitation Center of Beijing Rehabilitation Hospital between April 2020 and July 2024. A total of 215 patients with idiopathic PD were admitted to the rehabilitation ward. Of these, 122 patients met all inclusion criteria and were included in the final analysis. The remaining 93 patients were excluded for the following reasons: 75 had incomplete clinical or assessment data; 48 had a diagnosis of dementia or severe cognitive impairment that prevented reliable evaluation; nine had comorbidities such as stroke, fractures, or severe osteoarthritis that interfered with motor assessment or training; seven were subject to medication adjustments; and two were diagnosed with atypical or secondary Parkinsonism. This rigorous selection process ensured the quality and consistency of the analyzed sample. PWP were classified into two groups, PDA+ (PD with apathy) and PDA− (PD without apathy), based on their Modified Apathy Evaluation Scale (MAES) scores using a predefined cutoff for clinically significant apathy (see Table 1). The predefined cutoff of MAES > 14 to define apathy followed prior validation work (Starkstein et al. 1992; Thobois et al. 2013) and was prespecified before analysis. Inclusion criteria included (1) idiopathic PD diagnosed by a neurologist according to the Movement Disorder Society criteria (Postuma et al. 2015), with Hoehn–Yahr (H‐Y) stage ≤ 3 and (2) an ability to cooperate with treatment and evaluation. The exclusion criteria were (1) atypical or secondary PD; (2) dementia; (3) severe visual and hearing impairment; (4) other diseases, such as fractures, lumbar disc herniation, and osteoarthritis that affected walking ability; (5) other neuropsychiatric disorders or surgical history of brain; and (6) changes in antiparkinsonian drug therapy during the whole study period.

**TABLE 1:** Baseline characteristics of participants before MIRT.

Items	PDA+(*n* = 47)	PDA−(*n* = 75)	*p*
Gender (male, *n* [%])	19 (40.43)	36 (48.00)	0.413
H‐Y stage median (IQR)	2.00 (1.00)	2.00 (1.00)	0.465
Disease duration (year) (median [IQR])	6.00 (4.00)	6.00 (4.00)	0.422
Age (year) (mean± SD)	61.89 ± 5.38	61.08 ± 6.87	0.497
LEDD (mg)(mean± SD)	502.07 ± 225.91	528.31 ± 218.68	0.525
MDS‐UPDRS III (median [IQR])	27.00 (19.00)	30.00 (18.00)	0.714
M‐PAS (median [IQR])	86.00 (5.00)	84.00 (12.00)	0.096
10MWT‐comfortable gait speed (m/s) (median [IQR])	1.23 (0.00)	1.23 (0.00)	0.577
10MWT‐fast gait speed (m/s) (median [IQR])	1.69 (0.00)	1.68 (0.00)	0.391
FTSTS (s) (median [IQR])	9.27 (3.00)	10.44 (3.00)	0.011*
TUG (s) (median [IQR])	8.27 (2.00)	8.64 (2.00)	0.046*
MoCA (median [IQR])	26.00 (4.00)	27.00 (4.00)	0.419
HARS (median [IQR])	9.00 (10.00)	10.00 (9.00)	0.852
HAMD (median [IQR])	9.00 (8.00)	7.00 (8.00)	0.093
MAES (median [IQR])	20.00 (6.00)	9.00 (7.00)	< 0.001*
PFS‐16 (median [IQR])	43.00 (26.00)	44.00 (24.00)	0.823

Abbreviations: 10MWT, 10‐Meter Walk; FTSTS, Five Times Sit to Stand; HAMD, Hamilton Depression Scale; HARS, Hamilton Anxiety Rating Scale; H‐Y, Hoehn–Yahr stage; LEDD, L‐dopa equivalent dose; MAES, Modified Apathy Evaluation Scale; MDS‐UPDRS III, Movement Disorder Society–Unified Parkinson's Disease Rating Scales III; MoCA, Montreal Cognitive Assessment; M‐PAS, Modified Parkinson Activity Scale; *n*, number of patients; PFS, Parkinson Fatigue Scale; TUG, Timed Get Up and Go.

*, *p* < 0.05.

The Beijing Rehabilitation Hospital of Capital Medical University ethics review board approved this study. The written consent was obtained from all subjects in accordance with the Declaration of Helsinki.

### 2.2 Study Protocol

All participants engaged in the brief 2‐week MIRT program previously described in our prior research, which encompassed four daily rehabilitation sessions conducted in a hospital setting (Chen et al. 2021). The MIRT regimen included four sessions per day, 5 days a week, over a total duration of 2 weeks. Each individual session lasted between 30 and 60 min. The first session involved personalized physical therapy, while the second focused on balance and gait exercises. The third session was dedicated to aerobic training, and the fourth involved speech therapy. Evaluations were performed by the same physiotherapist and neurologist upon admission and discharge. All evaluations were carried out during the ON state, which is defined as 1–2 h post‐medication. The rehabilitation protocol was standardized across all participants. Both pre‐ and posttreatment assessments were conducted within 1–2 days of admission and discharge, respectively, ensuring a consistent 2‐week intervention period and minimizing variability in treatment exposure. All participants maintained a stable dopaminergic medication regimen throughout the 2‐week MIRT intervention, with no changes in levodopa equivalent daily doses (LEDD) or other pharmacological adjustments during this period.

### 2.3 Collection of Demographic Information

Demographic information includes sex, age, disease duration, and LEDD. The LEDD was calculated based on a previous report (Tomlinson et al. 2010).

### 2.4 Motor Function Assessment

Motor symptoms were assessed utilizing several established scales, including the Movement Disorder Society Unified PD Rating Scale part III (MDS‐UPDRS III) (Goetz et al. 2008), the Modified Parkinson Activity Scale (M‐PAS) (Keus et al. 2009), Five Times Sit to Stand (FTSTS) (Duncan et al. 2011), Timed Up and Go (TUG) (Podsiadlo and Richardson 1991), and 10‐Meter Walking (10MW) (Lang et al. 2016).

### 2.5 Non‐Motor Function Assessment

Apathy was evaluated by MAES (Starkstein and Mayberg 1992), the scale has 14 items, and the subjects are evaluated from three aspects: cognition, emotion, and behavior. A score of > 14 is defined as apathetic emotion, and the higher the score is, the more severe the degree of apathetic emotion. Furthermore, cognitive function was evaluated by the Montreal Cognitive Assessment (MoCA) (Nasreddine et al. 2005), depression was evaluated by the Hamilton Depression Scale (HAMD) (Hamilton 1960), anxiety was evaluated by the Hamilton Anxiety Rating Scale (HARS) (Hamilton 1959), and fatigue was evaluated by the Parkinson Fatigue Scale (PFS⁃16) (Brown et al. 2005) at baseline. It is crucial to emphasize that the MAES assesses a patient's level of apathy over the preceding 4 weeks (Robert et al. 2009). In this research, which incorporated a 2‐week inpatient rehabilitation phase, the apathy scores recorded upon admission were regarded as indicative of a stable baseline state. No additional MAES evaluations were carried out at the time of discharge since the short duration of hospitalization did not permit the detection of significant changes in apathy within the same individual. This methodology is consistent with other non‐motor assessments designed to evaluate relatively stable behavioral or emotional states, rather than to capture short‐term fluctuations.

### 2.6 Statistical Analysis

Data that follow a normal distribution are represented as the mean ± standard deviation, whereas data that do not conform to a normal distribution are presented as the median along with the interquartile range. The normality of the data was assessed utilizing the Shapiro–Wilk test. To compare the data obtained prior to and following the intervention, the Wilcoxon matched‐pairs signed‐ranks test was employed. Between‐group comparisons for H–Y stage, disease duration, MoCA, HAMD, HARS, MAES, PFS⁃16, MDS‐UPDRS III, M‐PAS, 10MWT‐comfortable gait speed, 10MWT‐fast gait speed, FTSTS, and TUG were assessed using the Mann–Whitney *U* test, while age and LEDD were assessed using the independent samples *t*‐test at baseline. The sex ratio was compared using the chi‐squared test. To correct the baseline imbalance factor (for FTSTS and TUG), multiple linear regression was used. To evaluate the robustness of the findings, we performed additional analyses treating MAES as both a continuous and a dichotomous variable (cutoff = 14). Sensitivity analyses were further conducted by shifting the cutoff value (e.g., MAES > 13 or > 15) to assess the stability of group classifications and the impact of borderline cases. There was statistical significance if *p* < 0.05. All analyses were performed using SPSS Statistics version 27.0 software (IBM, Armonk, NY, USA).

## 3 Results

### 3.1 Characteristics of the PWP in Each Group

The PDA+ group comprised 19 male and 28 female patients, with a mean age of 61.89 ± 5.38 years (range 51–72 years), and median H‐Y stage of 2.00 (interquartile range: 1.00; stage 1.5, *n* = 5; stage 2, *n* = 24; stage 2.5, *n* = 8; and stage 3, *n* = 10). The PDA− group had 36 male and 39 female patients, with a mean age of 61.08 ± 6.87 years (range 43–77 years) and median H‐Y stage of 2.00 (interquartile range: 1.00; stage 1, *n* = 2; stage 1.5, *n* = 12; stage 2, *n* = 33; stage 2.5, *n* = 15; and stage 3, *n* = 13). A significantly higher MAES score was observed in the PDA+ group (20.00 [IQR 6.00]) compared with the PDA− group (9.00 [IQR 7.00]; *p* < 0.001).There were no differences in gender, H‐Y stage, age, disease duration, mean LEDD, MDS‐UPDRS III, M‐PAS, 10MWT‐comfortable gait speed, 10MWT‐fast gait speed, MoCA, HARS, HAMD, and PFS‐16 between the two groups at baseline (*p* > 0.05). However, FTSTS and TUG differed significantly at baseline (*p* < 0.05) and was therefore used as an independent variable in the linear regression correction analysis (Table 1).

### 3.2 Motor Function Outcomes After MIRT

After 2 weeks of MIRT, PDA− group had significant improvements in MDS‐UPDRS III (*β* = –5.11, 95% CI: –6.28 to –3.94, *p* < 0.001), M‐PAS (*β* = 2.16, 95% CI: 0.61–3.71, *p* < 0.001), FTSTS (*β* = –0.41, 95% CI: –0.79 to –0.03, *p* = 0.011), and TUG (β = –0.78, 95% CI: –1.50 to –0.05, *p* < 0.001; Table 2). There were no significant changes in the PDA+ group across all outcome measures, including MDS‐UPDRS III (*β* = –2.34, 95% CI: –4.21 to –0.47, *p* = 0.250), FTSTS (*β* = 0.12, 95% CI: –0.37 to 0.61, *p* = 0.645), and TUG (*β* = –0.15, 95% CI: –0.49 to 0.18, *p* = 0.522; Table 2). For the PDA+ group, comparison of change scores before and after intervention showed that the PDA− group had significantly greater improvements in MDS‐UPDRS III (between‐group difference: *β* = –2.77, 95% CI: –5.08 to –0.45, *p* = 0.018), FTSTS (*β* = –0.53, 95% CI: –1.05 to –0.01, *p* = 0.046), and TUG (*β* = –0.63, 95% CI: –1.23 to –0.02, *p* = 0.042; Table 2).

**TABLE 2:** Motor function assessment before and after training.

Items	PDA+(*n* = 47)				PDA−(*n* = 75)				Δ *p* value between groups
	Pre‐MIRT	Post‐MIRT	Δ	*p*	Pre‐MIRT	Post‐MIRT	Δ	*p*	*p*
MDS‐UPDRS III	27.00 (19.00)	26.00 (14.00)	−2.34 (−4.21, −0.47)	0.250	30.00 (18.00)	23.00 (18.00)	−5.11 (−6.28, −3.94)	< 0.001**	0.018*
M‐PAS	86.00 (5.00)	86.00 (5.00)	−2.72 (−7.56, 2.11)	0.075	84.00 (12.00)	85.00 (7.00)	2.16 (0.61, 3.71)	< 0.001**	0.061
10MWT‐Comfortable gait speed (m/s)	1.23 (0.00)	1.30 (0.00)	0.33 (−0.01, 0.07)	0.079	1.23 (0.00)	1.28 (0.00)	0.29 (−0.01, 0.06)	0.084	0.736
10MWT‐Fast gait speed (m/s)	1.69 (0.00)	1.72 (0.00)	0.02 (−0.05,0.12)	0.115	1.68 (0.00)	1.70 (0.00)	0.02 (−0.03,0.07)	0.339	0.799
FTSTS (s)	9.27 (3.00)	9.73 (3.00)	0.12 (−0.37, 0.61)	0.645	10.44 (3.00)	9.75 (3.00)	−0.41 (−0.79, −0.03)	0.011*	0.046*
TUG (s)	8.27 (2.00)	8.13 (2.00)	−0.15 (−0.49, 0.18)	0.522	8.64 (2.00)	8.44 (3.00)	−0.78 (−1.50, −0.05)	< 0.001**	0.042*

*Note*: Δ: Difference within groups before and after intervention expressed as difference and 95 % CI.

Abbreviations: 10MWT, 10‐Meter Walk; FTSTS, Five Times Sit to Stand; MDS‐UPDRS III, Movement Disorder Society–Unified Parkinson's Disease Rating Scales III; M‐PAS, Modified Parkinson Activity Scale; *n*, number of patients; TUG, Timed Get Up and Go.

*, *p* < 0.05; **, *p* < 0.01.

### 3.3 Multiple Linear Regression

**FIGURE 1:**
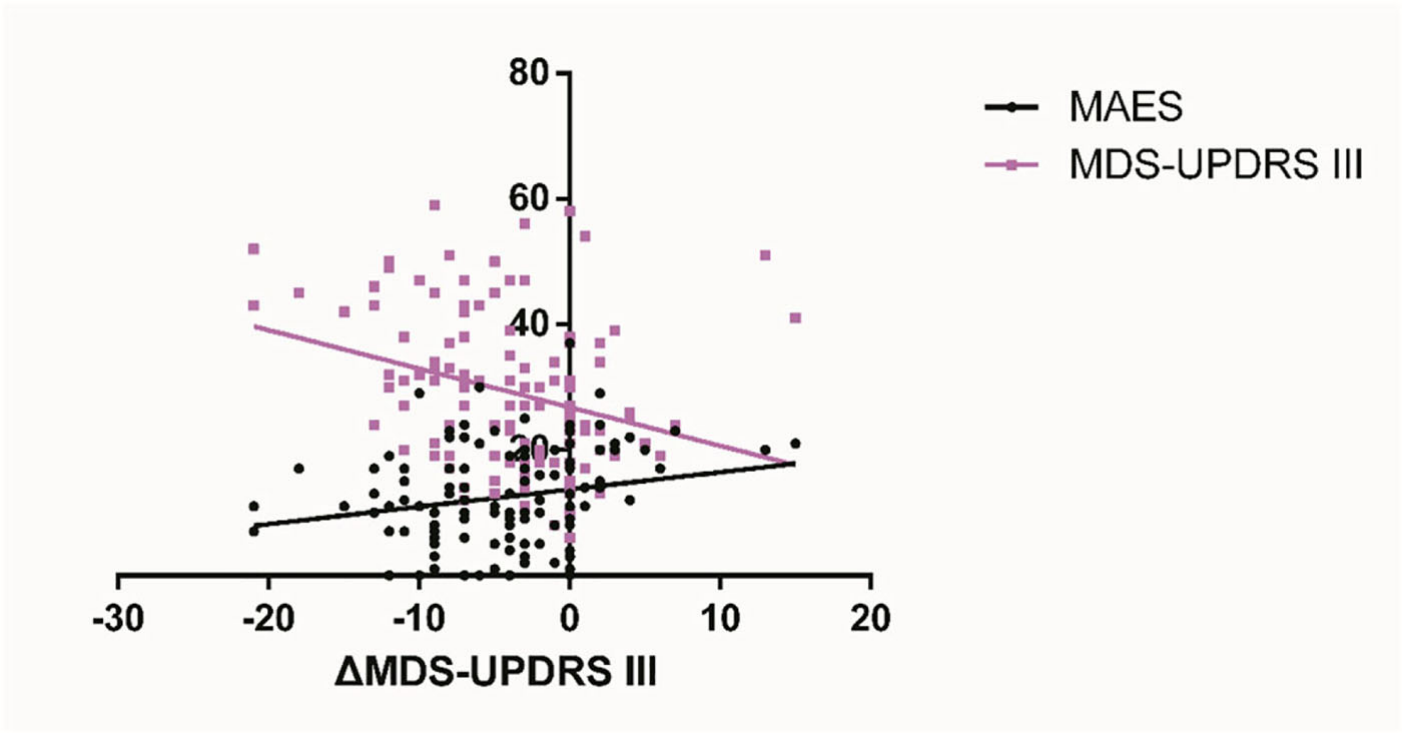
Relations between ΔMDS‐UPDRS III and MAES and MDS‐UPDRS III in PWP. ΔMDS‐UPDRS III: Changes in MDS‐UPDRS III after MIRT. MAES, Modified Apathy Evaluation Scale; MDS‐UPDRS III, Movement Disorder Society–Unified PD Rating Scales III; PWP, patients with Parkinson's disease.

After adjusting for baseline imbalanced factors using multiple linear regression, both MAES (*β* = 0.147, 95% CI: 0.017–0.277, *p* = 0.027) and baseline MDS‐UPDRS III score (*β* = –0.139, 95% CI: –0.221 to –0.057, *p* = 0.001) were identified as significant predictors of changes in MDS‐UPDRS III after training (Table 3). There were no significant associations for other covariates, including FTSTS (*β* = –0.253, 95% CI: –0.722 to 0.216, *p* = 0.288) and TUG (*β* = –0.015, 95% CI: –0.364 to 0.333, *p* = 0.930). At the same time, the MAES is positively correlated with △MDS‐UPDRS III (the change in MDS‐UPDRS III score from pretraining to post‐training)(Figure 1; Table 3), meaning that individuals with higher apathy may show less improvement in MDS‐UPDRS III after training. Dichotomous analyses of MAES scores showed that patients with higher baseline apathy exhibited significantly smaller improvements in MDS‐UPDRS III after MIRT (Table S1). Similarly, sensitivity analyses redefining the apathy cutoff (using MAES > 13 or > 15) produced consistent findings, with the higher apathy subgroup persistently showing attenuated motor improvement (Tables S2 and S3).

**TABLE 3:** Multiple linear regression of baseline MAES scores (continuous) and ΔMDS‐UPDRS III.

	Coefficients						
	Unstandardized coefficients		Standardized coefficients				
Model	*β*	SEM	*β′*	*t*	*p*	95.0% confidence interval for *β*	*R*2
Constant	0.903	2.798	—	0.323	0.748	(−4.639 to 6.445)	0.145
MAES	0.147	0.066	0.197	2.235	0.027*	(0.017 to 0.277)	
MDS‐UPDRS III	−0.139	0.041	−0.291	−3.353	0.001**	(−0.221 to −0.057)	
FTSTS (s)	−0.253	0.237	−0.102	−1.068	0.288	(−0.722 to 0.216)	
TUG (s)	−0.015	0.176	−0.008	−0.088	0.930	(−0.364 to 0.333)	

*Note*: Multiple linear regression was used, and the basis for single‐factor variables entering the regression model was *p* < 0.05. ΔMDS‐UPDRS III: Changes in MDS‐UPDRS III after MIRT; Dependent variable: ΔMDS‐UPDRS III.

Abbreviations: MAES, Modified Apathy Evaluation Scale; MDS‐UPDRS III, Movement Disorder Society–Unified PD Rating Scales III; FTSTS, Five Times Sit to Stand; TUG, Timed Get Up and Go.

*, p < 0.05; **, *p* < 0.01.

## 4 Discussion

Following a 2‐week MIRT intervention, the PDA− group demonstrated significant improvements in MDS‐UPDRS III, FTSTS, and TUG assessments. In contrast, no statistically significant changes were observed in the PDA+ group for any of these measures. Specifically, the lack of significant improvement in FTSTS and TUG within the PDA+ cohort may be attributable to their superior baseline motor function. This baseline advantage may partly attenuate observable post‐rehabilitation gains in the PDA+ group due to potential ceiling effects, and underscores the importance of baseline adjustment and stratified analyses in future studies. Accordingly, the smaller short‐term gains in the PDA+ group should not be interpreted as being solely attributable to apathy. Although baseline motor performance was adjusted for, ceiling effects and potential nonlinear baseline–change relationships cannot be fully excluded in this retrospective dataset. Despite these considerations, this study found that short‐term improvements in MDS‐UPDRS III scores following MIRT were more pronounced in the PDA− group compared to the PDA+ group, suggesting that individuals with higher levels of apathy may benefit less from motor training. Overall, our results indicate an association between higher baseline apathy (MAES) and smaller short‐term motor gains after MIRT. The findings were further corroborated by consistent results across both the dichotomous and continuous models of the MAES, as well as through sensitivity analyses using alternative cutoff values (using MAES > 13 and > 15). The consistent correlation between higher baseline apathy and diminished motor improvement, regardless of the analytical approach or cutoff definition employed, suggests that this relationship is not merely an outcome of the specific grouping threshold. These results enhance the credibility of apathy as an independent predictor of rehabilitation outcomes in PWP, highlighting that even minor fluctuations in the severity of apathy may affect the potential for motor recovery. From a clinical perspective, these findings are crucial. When designing rehabilitation programs, it is essential to evaluate the presence of apathy in patients. For those exhibiting marked apathy, in addition to conventional motor training, incorporating psychological incentives or other targeted interventions may be necessary to enhance patient engagement and improve training outcomes. Furthermore, this observation offers a novel perspective for further investigating the relationship between dopaminergic function and non‐motor symptoms in PD rehabilitation.

Although apathy is commonly observed in PWP, it remains one of the most intricate and least understood symptoms of the condition (Mele et al. 2020). Apathy in PD is multidimensional, stemming from dysfunctions in various neural systems (Pagonabarraga et al. 2015). This complexity is exemplified by its three distinct subtypes: emotional‐affective, cognitive, and auto‐activation deficits (Levy and Dubois 2006). A consideration of the multidimensional nature of apathy is essential for understanding its major components in PD. During the early and fluctuating stages of PD, apathy may predominantly manifest as a motivational disorder that in certain cases and depending on subtype can be partially alleviated by dopaminergic therapy, whereas other subtypes may be less responsive. However, in later stages, apathy becomes more intricately associated with cognitive decline, executive dysfunction, and the onset of PD dementia (Béreau et al. 2023). As the study cohort comprised predominantly patients in the early to moderate stages of disease with the capacity to engage in an inpatient rehabilitation program, interpretation of the findings must account for the representativeness and generalizability of this specific clinical population. All patients received standardized MIRT interventions during hospitalization with strict adherence to the prescribed training frequency and duration. Although all patients completed the full 2‐week MIRT program under supervision, detailed adherence metrics were not systematically recorded in this retrospective dataset. Taken together, our findings may reflect an association between apathy and attenuated motor improvement following MIRT, although causal inferences cannot be made due to the observational design. Within this clinical context, it is plausible that even when patients complete the scheduled sessions and training frequency is consistent, apathy may still be associated with smaller rehabilitation gains, thereby emerging as an important correlate of rehabilitation outcomes. Notably, although motivation and consistent attendance are fundamental prerequisites for maximizing rehabilitation benefits, apathy may manifest not in session nonattendance but as suboptimal engagement. Because we did not systematically capture session‐level engagement or adherence metrics in this retrospective dataset, we can only hypothesize that such suboptimal engagement might involve diminished effort, reduced sustained attention, or inadequate effective practice intensity during therapeutic activities, which could attenuate functional gains. Future studies should consider quantifying adherence more precisely and formally testing mediation pathways, as these factors may represent potential mechanisms linking apathy to rehabilitation outcomes. Beyond neurobiological mechanisms, apathy may also impair engagement in health‐promoting behaviors and reduce adherence to rehabilitation, thereby limiting potential functional gains.

The association between apathy and rehabilitation outcomes may involve multiple pathways, including neurobiological, psychological, and social‐behavioral factors. Apathy in PD is associated with widespread dopaminergic dysfunction. Le Heron et al. (2018) proposed that apathy arises from impaired functioning within the fronto‐striatal network, which regulates three critical components of motivated behavior: (i) initiating actions, (ii) maintaining actions, and (iii) learning from outcomes to evaluate the value of behaviors. However, some studies suggest that apathy in PD is unlikely to result solely from the dysfunction of a single brain circuit, indicating that multiple neural networks contribute to this complex symptom (Gilmour et al. 2024). Gilmour et al. (2024) found that in a value‐based decision‐making task, PWP exhibited impaired outcome encoding, with apathetic patients showing significantly reduced activity in the left ventromedial prefrontal cortex. During exploratory decisions, non‐apathetic PWP recruited additional brain regions as compensatory mechanisms, while apathetic patients demonstrated insufficient activation in the thalamus/midbrain and parietal‐occipital areas. Notably, right thalamic activity correlated with the severity of apathy. These findings suggest that deficits in cognitive control and information integration may underlie apathy and potentially impact rehabilitation outcomes. Similar disruptions have been observed in other conditions. For example, post‐stroke apathetic lesions affecting the frontal‐striatal‐thalamic circuit can impair patients' ability to integrate action goals with environmental feedback during rehabilitation (Tay et al. 2021). In PD, an imbalance in reward and effort evaluation leads to reduced reward sensitivity and heightened effort sensitivity (Salamone et al. 2016; Husain and Roiser 2018), which may be more pronounced in apathetic patients. This imbalance results in diminished awareness of personal progress and an excessive focus on avoiding failure, ultimately undermining sustained engagement. Research indicates that combining noninvasive brain stimulation with cognitive training can partially restore impaired feedback processing, thereby enhancing rehabilitation outcomes (Amaya‐Pascasio et al. 2024). Hoy et al. (2024) identified distinct neural signatures of reward and effort in decision‐making through intracranial recordings in PWP. They observed that β power in the basal ganglia decreases with increasing effort, while θ power in the prefrontal cortex increases following reward delivery. Importantly, the prefrontal cortex stimulation enhanced reward sensitivity, reduced effort sensitivity, and increased task acceptance, underscoring its causal role in motivation. These findings suggest that targeting the prefrontal cortex could refine neuromodulation strategies for treating motivational deficits in neurological disorders. Consistent with previous literature indicating that apathy in PWP is associated with deficits in motivation and executive function, our study further investigates the impact of apathy on rehabilitation effectiveness. Prior research has shown that apathy in PD is closely linked to diminished dopaminergic function and impairments in the prefrontal cortex and basal ganglia circuits—neural pathways that also play a critical role in reward processing and motor control. However, because we did not collect neuroimaging, neurophysiological, or biomarker measures, our data cannot inform circuit‐level mechanisms, and any mechanistic interpretation should be considered speculative and hypothesis‐generating. Therefore, we hypothesize that apathy may attenuate patients’ responses to positive reinforcement during rehabilitation training, thereby diminishing training quality and ultimately affecting overall therapeutic efficacy. This hypothesis warrants direct testing in future prospective studies incorporating neurobiological measures.

Although apathy can occur in isolation, it frequently co‐occurs with other neuropsychiatric symptoms in early PD, such as cognitive impairment, anxiety, depression, and fatigue. Martin et al. (2020) conducted a 2‐year follow‐up study on 104 PWP and found a significant negative correlation between apathy and cognitive function, suggesting that apathy may serve as an early behavioral marker of cognitive decline in PWP. The development of apathy is closely linked to disruptions in the frontal‐striatal network, which can result in cognitive decline, particularly in executive function (D'Iorio et al. 2018; Wen et al. 2022). The pathogenesis of anxiety, depression, fatigue, and apathy in PWP is associated with dopaminergic neuronal deficits (Tessitore et al. 2016; Wen et al. 2016). Research indicates that while apathy, depression, anxiety, and fatigue are common and interrelated in PWP, confirmatory factor analysis shows that pure apathy can be distinguished from these other symptoms (Ineichen and Baumann‐Vogel 2021). In our study, at baseline, there were no significant differences between the two groups in MoCA, HARS, HAMD, and PFS‐16 scores, thereby minimizing the influence of confounding factors.

This study has several limitations. First, the relatively small sample size and monocentric, retrospective design may limit the generalizability of the findings, especially given the exclusion of patients with H–Y stage greater than 3 and those with comorbidities such as hearing or severe mobility impairments. Future multicenter prospective studies including broader disease stages are warranted to validate the observed association. Second, apathy was assessed only once at baseline using the MAES, and no posttreatment reassessment was available. This single‐timepoint design meant that within‐subject fluctuations could not be captured, and longitudinal models such as GEEs or GLMMs could not be applied. Future studies should therefore include repeated apathy assessments to better understand its dynamic course. Third, although we adjusted for selected baseline imbalances using multiple linear regression, not all potential confounders were fully incorporated. Cognitive status, depressive symptoms, anxiety, fatigue, and other motor and non‐motor features may still have influenced outcomes. In addition, adherence and psychosocial mediators were not recorded. Although all patients completed the supervised 2‐week MIRT program, detailed engagement metrics such as repetitions performed, perceived effort, or satisfaction with care were not systematically quantified. Other factors including comorbidities or pain may also have affected both apathy and rehabilitation outcomes. Fourth, the classification of apathy was based on a validated but arbitrary MAES cutoff of 14, which may oversimplify the continuous nature of the construct. Fifth, although LEDD remained stable during the intervention, pharmacological effects may vary between individuals and could act as confounders or effect modifiers. Future work should model these explicitly. Finally, only physical function outcomes were assessed before and after rehabilitation. Data on mood, quality of life, or neuropsychiatric symptoms after training were not available.

## 5 Conclusion

In conclusion, this study demonstrates that apathy is significantly associated with reduced motor improvement following multidisciplinary intensive rehabilitation in PWP. Higher MAES scores were related to less favorable changes in MDS‐UPDRS III after adjustment for baseline motor performance and other covariates, supporting apathy as a clinically relevant correlate of short‐term rehabilitation response. These findings highlight the importance of assessing motivational states when planning individualized rehabilitation strategies. Future research should aim to investigate the longitudinal dynamics of apathy, its impact on treatment adherence, and its underlying neurobiological mechanisms to optimize rehabilitation outcomes for this population.

## Author Contributions

**Yonghong Liu**: methodology, data curation. **Jinping Fang**: methodology, data curation, writing – original draft. **Keke Chen**: methodology, data curation. **Ruidan Wang**: data curation. **Xia An**: data curation. **Detao Meng**: data curation. **Zhenzhen Li**: data curation. **Hongjiao Yan**: data curation. **Cuiping Xue**: data curation. **Tingting Hou**: data curation. **Hongyu Zhang**: data curation. **Yi Zhen**: data curation. **Boyan Fang**: data curation, methodology, conceptualization, writing – review and editing, project administration. **Yixuan Wang**: data curation.

## Funding

This study was supported by the National Key R&D Program of China (No. 2022YFC3602603) for Boyan Fang, the Science and Technology Development Fund of Beijing Rehabilitation Hospital, Capital Medical University (2020‐069 for Boyan Fang), and Science and Technology Development Project of Beijing Rehabilitation Hospital Affiliated to Capital Medical University (2023R‐03 for Detao Meng). The funding body had no role in protocol design, statistical analysis, and manuscript preparation.

## Ethics Statement

This study was approved by the Ethics Committee of the Beijing Rehabilitation Hospital of Capital Medical University (Approval No. 2021bkky‐001). Before enrollment, a written informed consent was obtained from all individual participants for the publication of their data, including any accompanying images.

## Conflicts of Interest

The authors declare no conflicts of interest.

## Supporting information




**Supplementary Materials**: brb371480‐sup‐0001‐SuppMat.docx

## Data Availability

The data supporting the findings of this study are available on request from the corresponding author. The data are not publicly available due to privacy or ethical restrictions.

## References

[brb371480-bib-0001] Afshari, M. , A. Yang , and D. Bega . 2017. “Motivators and Barriers to Exercise in Parkinson's Disease.” Journal of Parkinson's Disease 7: 703–711. 10.3233/JPD-171173 29103050

[brb371480-bib-0002] Amaya‐Pascasio, L. , J. García‐Pinteño , A. Sánchez‐Kuhn , et al. 2024. “Neuromodulation of Executive Dysfunction in Patients With Acute Stroke Using Transcranial Direct Current Stimulation: Study Protocol for a Triple‐Blind, Randomized Sham‐Controlled Trial.” Cerebrovascular Diseases 53: 335–345. 39250901 10.1159/000531860

[brb371480-bib-0003] Béreau, M. , V. Van Waes , M. Servant , E. Magnin , L. Tatu , and M. Anheim . 2023. “Apathy in Parkinson's Disease: Clinical Patterns and Neurobiological Basis.” Cells 12: 1599. 37371068 10.3390/cells12121599 PMC10297386

[brb371480-bib-0004] Bloem, B. R. , N. M. De Vries , and G. Ebersbach . 2015. “Nonpharmacological Treatments for Patients With Parkinson's Disease.” Movement Disorders 30: 1504–1520. 26274930 10.1002/mds.26363

[brb371480-bib-0005] Bloem, B. R. , M. S. Okun , and C. Klein . 2021. “Parkinson's Disease.” Lancet 397: 2284–2303. 33848468 10.1016/S0140-6736(21)00218-X

[brb371480-bib-0006] Brötz, D. 2013. “Effectiveness of Intensive Inpatient Rehabilitation Treatment on Disease Progression in Parkinsonian Patients: A Randomized Controlled Trial With 1‐Year Follow‐Up.” Physioscience 9: 38–38. 10.1177/1545968311416990 21844282

[brb371480-bib-0007] Brown, R. G. , A. Dittner , L. Findley , and S. C. Wessely . 2005. “The Parkinson Fatigue Scale.” Parkinsonism & Related Disorders 11: 49–55. 15619463 10.1016/j.parkreldis.2004.07.007

[brb371480-bib-0008] Chen, K.‐K. , Z.‐H. Jin , L. Gao , et al. 2021. “Efficacy of Short‐Term Multidisciplinary Intensive Rehabilitation in Patients With Different Parkinson's Disease Motor Subtypes: A Prospective Pilot Study With 3‐Month Follow‐Up.” Neural Regeneration Research 16: 1336. 33318414 10.4103/1673-5374.301029 PMC8284270

[brb371480-bib-0009] D'Iorio, A. , G. Maggi , C. Vitale , L. Trojano , and G. Santangelo . 2018. ““Pure Apathy” and Cognitive Dysfunctions in Parkinson's Disease: A Meta‐Analytic Study.” Neuroscience & Biobehavioral Reviews 94: 1–10. 30114389 10.1016/j.neubiorev.2018.08.004

[brb371480-bib-0010] Dorsey, E. R. , and B. R. Bloem . 2018. “The Parkinson Pandemic—A Call to Action.” JAMA Neurology 75: 9. 29131880 10.1001/jamaneurol.2017.3299

[brb371480-bib-0011] Duncan, R. P. , A. L. Leddy , and G. M. Earhart . 2011. “Five Times Sit‐to‐Stand Test Performance in Parkinson's Disease.” Archives of Physical Medicine and Rehabilitation 92: 1431–1436. 21878213 10.1016/j.apmr.2011.04.008 PMC3250986

[brb371480-bib-0012] Ferrazzoli, D. , P. Ortelli , I. Zivi , et al. 2018. “Efficacy of Intensive Multidisciplinary Rehabilitation in Parkinson's Disease: A Randomised Controlled Study.” Journal of Neurology, Neurosurgery & Psychiatry 89: 828–835. 29321141 10.1136/jnnp-2017-316437 PMC6204945

[brb371480-bib-0013] Gilmour, W. , G. Mackenzie , M. Feile , et al. 2024. “Impaired Value‐Based Decision‐Making in Parkinson's Disease Apathy.” Brain 147: 1362–1376. 38305691 10.1093/brain/awae025 PMC10994558

[brb371480-bib-0014] Goetz, C. G. , B. C. Tilley , S. R. Shaftman , et al. 2008. “Movement Disorder Society‐Sponsored Revision of the Unified Parkinson's Disease Rating Scale (MDS‐UPDRS): Scale Presentation and Clinimetric Testing Results.” Movement Disorders 23: 2129–2170. 19025984 10.1002/mds.22340

[brb371480-bib-0015] Hama, S. , H. Yamashita , M. Shigenobu , et al. 2007. “Depression or Apathy and Functional Recovery After Stroke.” International Journal of Geriatric Psychiatry 22: 1046–1051. 17702056 10.1002/gps.1866

[brb371480-bib-0016] Hamilton, M. 1959. “The Assessment of Anxiety States by Rating.” British Journal of Medical Psychology 32: 50–55. 13638508 10.1111/j.2044-8341.1959.tb00467.x

[brb371480-bib-0017] Hamilton, M. 1960. “A Rating Scale for Depression.” Journal of Neurology, Neurosurgery & Psychiatry 23: 56–62. 14399272 10.1136/jnnp.23.1.56 PMC495331

[brb371480-bib-0018] Hinkle, J. T. , K. Perepezko , L. L. Gonzalez , K. A. Mills , and G. M. Pontone . 2021. “Apathy and Anxiety in De Novo Parkinson's Disease Predict the Severity of Motor Complications.” Movement Disorders Clinical Practice 8: 76–84. 33426161 10.1002/mdc3.13117 PMC7780944

[brb371480-bib-0019] Hoy, C. W. , C. De Hemptinne , S. S. Wang , et al. 2024. “Beta and Theta Oscillations Track Effort and Previous Reward in the Human Basal Ganglia and Prefrontal Cortex During Decision Making.” Proceedings of the National Academy of Sciences 121: e2322869121. 10.1073/pnas.2322869121 PMC11295073 39047043

[brb371480-bib-0020] Husain, M. , and J. P. Roiser . 2018. “Neuroscience of Apathy and Anhedonia: A Transdiagnostic Approach.” Nature Reviews Neuroscience 19: 470–484. 29946157 10.1038/s41583-018-0029-9

[brb371480-bib-0021] Ineichen, C. , and H. Baumann‐Vogel . 2021. “Deconstructing Apathy in Parkinson's Disease: Challenges in Isolating Core Components of Apathy From Depression, Anxiety, and Fatigue.” Frontiers in Neurology 12: 720921. 34512530 10.3389/fneur.2021.720921 PMC8427284

[brb371480-bib-0022] Keus, S. H. J. , A. Nieuwboer , B. R. Bloem , G. F. Borm , and M. Munneke . 2009. “Clinimetric Analyses of the Modified Parkinson Activity Scale.” Parkinsonism & Related Disorders 15: 263–269. 18691929 10.1016/j.parkreldis.2008.06.003

[brb371480-bib-0023] Lang, J. T. , T. O. Kassan , L. L. Devaney , C. Colon‐Semenza , and M. F. Joseph . 2016. “Test‐Retest Reliability and Minimal Detectable Change for the 10‐Meter Walk Test in Older Adults with Parkinson's Disease.” Journal of Geriatric Physical Therapy 39: 165–170. 26428902 10.1519/JPT.0000000000000068

[brb371480-bib-0024] Langeskov‐Christensen, M. , E. Franzén , L. Grøndahl Hvid , and U. Dalgas . 2024. “Exercise as Medicine in Parkinson's Disease.” Journal of Neurology, Neurosurgery & Psychiatry 95: 1077–1088. 38418216 10.1136/jnnp-2023-332974

[brb371480-bib-0025] Le Heron, C. , M. A. J. Apps , and M. Husain . 2018. “The Anatomy of Apathy: A Neurocognitive Framework for Amotivated Behaviour.” Neuropsychologia 118: 54–67. 28689673 10.1016/j.neuropsychologia.2017.07.003 PMC6200857

[brb371480-bib-0026] Levy, R. 2012. “Apathy: A Pathology of Goal‐Directed Behaviour. A New Concept of the Clinic and Pathophysiology of Apathy.” Revue Neurologique 168: 585–597. 22921248 10.1016/j.neurol.2012.05.003

[brb371480-bib-0027] Levy, R. , and B. Dubois . 2006. “Apathy and the Functional Anatomy of the Prefrontal Cortex–Basal Ganglia Circuits.” Cerebral Cortex 16: 916–928. 16207933 10.1093/cercor/bhj043

[brb371480-bib-0028] Martin, G. P. , K. R. McDonald , D. Allsop , P. J. Diggle , and I. Leroi . 2020. “Apathy as a Behavioural Marker of Cognitive Impairment in Parkinson's Disease: A Longitudinal Analysis.” Journal of Neurology 267: 214–227. 31616991 10.1007/s00415-019-09538-z PMC6954881

[brb371480-bib-0029] Mele, B. , S. Van , J. Holroyd‐Leduc , Z. Ismail , T. Pringsheim , and Z. Goodarzi . 2020. “Diagnosis, Treatment and Management of Apathy in Parkinson's Disease: A Scoping Review.” BMJ Open 10: e037632. 10.1136/bmjopen-2020-037632 PMC7482451 32907903

[brb371480-bib-0030] Nasreddine, Z. S. , N. A. Phillips , V. Bédirian , et al. 2005. “The Montreal Cognitive Assessment, MoCA: A Brief Screening Tool for Mild Cognitive Impairment.” Journal of the American Geriatrics Society 53: 695–699. 15817019 10.1111/j.1532-5415.2005.53221.x

[brb371480-bib-0031] Pagonabarraga, J. , J. Kulisevsky , A. P. Strafella , and P. Krack . 2015. “Apathy in Parkinson's Disease: Clinical Features, Neural Substrates, Diagnosis, and Treatment.” Lancet Neurology 14: 518–531. 25895932 10.1016/S1474-4422(15)00019-8

[brb371480-bib-0032] Podsiadlo, D. , and S. Richardson . 1991. “The Timed “Up & Go”: A Test of Basic Functional Mobility for Frail Elderly Persons.” Journal of the American Geriatrics Society 39: 142–148. 1991946 10.1111/j.1532-5415.1991.tb01616.x

[brb371480-bib-0033] Postuma, R. B. , D. Berg , M. Stern , et al. 2015. “MDS Clinical Diagnostic Criteria for Parkinson's Disease: MDS‐PD Clinical Diagnostic Criteria.” Movement Disorders 30: 1591–1601. 26474316 10.1002/mds.26424

[brb371480-bib-0034] Robert, P. , C. U. Onyike , A. F. G. Leentjens , et al. 2009. “Proposed Diagnostic Criteria for Apathy in Alzheimer's Disease and Other Neuropsychiatric Disorders.” European Psychiatry 24: 98–104. 19201579 10.1016/j.eurpsy.2008.09.001

[brb371480-bib-0035] Salamone, J. D. , S. E. Yohn , L. López‐Cruz , N. San Miguel , and M. Correa . 2016. “Activational and Effort‐Related Aspects of Motivation: Neural Mechanisms and Implications for Psychopathology.” Brain 139: 1325–1347. 27189581 10.1093/brain/aww050 PMC5839596

[brb371480-bib-0036] Saleh, Y. , C. Le Heron , P. Petitet , et al. 2021. “Apathy in Small Vessel Cerebrovascular Disease Is Associated With Deficits in Effort‐Based Decision Making.” Brain 144: 1247–1262. 33734344 10.1093/brain/awab013 PMC8240747

[brb371480-bib-0037] Starkstein, S. E. , H. S. Mayberg , T. J. Preziosi , P. Andrezejewski , R. Leiguarda , and R. G. Robinson . 1992. “Reliability, Validity, and Clinical Correlates of Apathy in Parkinson's Disease.” Journal of Neuropsychiatry and Clinical Neurosciences 4: 134–139. 1627973 10.1176/jnp.4.2.134

[brb371480-bib-0038] Tay, J. , R. G. Morris , and H. S. Markus . 2021. “Apathy After Stroke: Diagnosis, Mechanisms, Consequences, and Treatment.” International Journal of Stroke 16: 510–518. 33527880 10.1177/1747493021990906 PMC8267086

[brb371480-bib-0039] Tessitore, A. , A. Giordano , R. De Micco , et al. 2016. “Functional Connectivity Underpinnings of Fatigue in “Drug‐Naïve” Patients With Parkinson's Disease.” Movement Disorders 31: 1497–1505. 27145402 10.1002/mds.26650

[brb371480-bib-0040] Thobois, S. , E. Lhommée , H. Klinger , et al. 2013. “Parkinsonian Apathy Responds to Dopaminergic Stimulation of D2/D3 Receptors With Piribedil.” Brain 136: 1568–1577. 23543483 10.1093/brain/awt067

[brb371480-bib-0041] Tomlinson, C. L. , R. Stowe , S. Patel , C. Rick , R. Gray , and C. E. Clarke . 2010. “Systematic Review of Levodopa Dose Equivalency Reporting in Parkinson's Disease.” Movement Disorders 25: 2649–2653. 21069833 10.1002/mds.23429

[brb371480-bib-0042] Trend, P. , J. Kaye , H. Gage , C. Owen , and D. Wade . 2002. “Short‐Term Effectiveness of Intensive Multidisciplinary Rehabilitation for People With Parkinson's Disease and Their Carers.” Clinical Rehabilitation 16: 717–725. 12428820 10.1191/0269215502cr545oa

[brb371480-bib-0043] Wen, M.‐C. , L. L. Chan , L. C. S. Tan , and E. K. Tan . 2016. “Depression, Anxiety, and Apathy in Parkinson's Disease: Insights From Neuroimaging Studies.” European Journal of Neurology 23: 1001–1019. 27141858 10.1111/ene.13002 PMC5084819

[brb371480-bib-0044] Wen, M.‐C. , A. Thiery , W.‐Y. I. Tseng , et al. 2022. “Apathy Is Associated With White Matter Network Disruption and Specific Cognitive Deficits in Parkinson's Disease.” Psychological Medicine 52: 264–273. 32524922 10.1017/S0033291720001907

