# Supplementary material for: The Correlation Between Apathy and the Efficacy of Rehabilitation in Patients With Parkinson's Disease: A Retrospective Observational Study
Source: Brain Behav. 2026 May 14;16(5):e71480. doi: 10.1002/brb3.71480 (PMC13176092; doi:10.1002/brb3.71480)
Supplement: Supplementary file 1 — Supplementary Materials: brb371480‐sup‐0001‐SuppMat.docx [file BRB3-16-e71480-s001.docx]

| Supplementary Table 1 Multiple linear regression of baseline MAES group (MAES ≤14 vs ＞14) and ΔMDS-UPDRS III |  | | *R^2^* |  | 0.148 |  |  |  |  | Multiple linear regression was used, and the basis for single-factor variables entering the regression model was P < 0.05. ΔMDS-UPDRS III: Changes in MDS-UPDRS III after MIRT; adependent variable: ΔMDS-UPDRS III; **P* < 0.05; ** *P* < 0.01. MAES: Modified Apathy Evaluation Scale;MDS-UPDRS III: Movement Disorder Society-Unified PD Rating Scales III; FTSTS: Five Times Sit to Stand; TUG: Timed Get Up and Go. |
| --- | --- | --- | --- | --- | --- | --- | --- | --- | --- | --- |
|  |  |  | 95.0% confidence interval for *β* |  | (–6.973 to 5.519) | (0.341 to 4.415) | (–0.212 to –0.049) | (–0.742 to 0. 187) | (–0.344 to 0.354) |  |
|  |  |  | *P* |  | 0.818 | **0.023*** | **0.002**** | 0.239 | 0.977 |  |
|  |  |  | *t* |  | -0.230 | 2.312 | -3.162 | -1.184 | 0.029 |  |
|  |  | Standardized coefficients | *β'* |  | - | 0.202 | -0.273 | -0.112 | 0.003 |  |
|  |  |  |  |  |  |  |  |  |  |  |
|  |  |  | SEM |  | 3.154 | 1.029 | 0.041 | 0.234 | 0.176 |  |
|  | Coefficients | Unstandardized coefficients | *β* |  | -0.727 | 2.378 | -0.130 | -0.278 | 0.005 |  |
|  |  | | Model |  | (Constant) | MAES group | MDS-UPDRS III | FTSTS (s) | TUG (s) |  |

| Supplementary Table 2 Multiple linear regression of baseline MAES group (MAES ≤13 vs ＞13) and ΔMDS-UPDRS III |  | | *R^2^* |  | 0.162 |  |  |  |  | Multiple linear regression was used, and the basis for single-factor variables entering the regression model was P < 0.05. ΔMDS-UPDRS III: Changes in MDS-UPDRS III after MIRT; adependent variable: ΔMDS-UPDRS III; **P* < 0.05; ** *P* < 0.01. MAES: Modified Apathy Evaluation Scale;MDS-UPDRS III: Movement Disorder Society-Unified PD Rating Scales III; FTSTS: Five Times Sit to Stand; TUG: Timed Get Up and Go. |
| --- | --- | --- | --- | --- | --- | --- | --- | --- | --- | --- |
|  |  |  | 95.0% confidence interval for *β* |  | (–7.860 to 4.656) | (0.741 to 4.745) | (–0.216 to –0.054) | (–0.715 to 0. 209) | (–0.326 to 0.367) |  |
|  |  |  | *P* |  | 0.748 | **0.008**** | **0.01*** | 0.281 | 0.907 |  |
|  |  |  | *t* |  | -0.507 | 2.713 | -3.302 | -1.083 | 0.117 |  |
|  |  | Standardized coefficients | *β'* |  | - | 0.237 | -0.283 | -0.102 | 0.011 |  |
|  |  |  |  |  |  |  |  |  |  |  |
|  |  |  | SEM |  | 3.160 | 1.011 | 0.041 | 0.233 | 0.175 |  |
|  | Coefficients | Unstandardized coefficients | *β* |  | -1.602 | 2.743 | -0.135 | -0.253 | 0.020 |  |
|  |  | | Model |  | (Constant) | MAES group | MDS-UPDRS III | FTSTS (s) | TUG (s) |  |

| Supplementary Table 3 Multiple linear regression of baseline MAES group (MAES ≤15 vs ＞15) and ΔMDS-UPDRS III |  | | *R^2^* |  | 0.162 |  |  |  |  | Multiple linear regression was used, and the basis for single-factor variables entering the regression model was P < 0.05. ΔMDS-UPDRS III: Changes in MDS-UPDRS III after MIRT; adependent variable: ΔMDS-UPDRS III; **P* < 0.05; ** *P* < 0.01. MAES: Modified Apathy Evaluation Scale;MDS-UPDRS III: Movement Disorder Society-Unified PD Rating Scales III; FTSTS: Five Times Sit to Stand; TUG: Timed Get Up and Go. |
| --- | --- | --- | --- | --- | --- | --- | --- | --- | --- | --- |
|  |  |  | 95.0% confidence interval for *β* |  | (–7.860 to 4.556) | (0.741 to 4.745) | (–0.216 to –0.054) | (–0.715 to 0. 209) | (–0.326 to 0.367) |  |
|  |  |  | *P* |  | 0.613 | **0.008**** | **0.01******** | 0.281 | 0.907 |  |
|  |  |  | *t* |  | -0.507 | 2.713 | -3.302 | -1.083 | 0.117 |  |
|  |  | Standardized coefficients | *β'* |  | - | 0.237 | -0.283 | -0.102 | -0.011 |  |
|  |  |  |  |  |  |  |  |  |  |  |
|  |  |  | SEM |  | 3.160 | 1.011 | 0.041 | 0.233 | 0.175 |  |
|  | Coefficients | Unstandardized coefficients | *β* |  | -1.602 | 2.743 | -0.135 | -0.253 | 0.020 |  |
|  |  | | Model |  | (Constant) | MAES group | MDS-UPDRS III | FTSTS (s) | TUG (s) |  |

**Plain Language Summary**

Apathy is commonly observed in patients with Parkinson's disease（PWP）, negatively influencing not only their mood and social functioning but also potentially compromising the effectiveness of rehabilitation training outcomes. Prior to this study, no investigations had examined the impact of apathy on motor rehabilitation outcomes in PWP. The primary objective of this research is to evaluate the influence of apathy on rehabilitation outcomes in PWP. A retrospective analysis was conducted on 122 PWP at Hoehn–Yahr stage ≤ 3 who underwent a two-week Multidisciplinary Intensive Rehabilitation Treatment (MIRT) program between April 2020 and July 2024. Based on the Modified Apathy Evaluation Scale (MAES), patients were categorized into two groups: Parkinson's disease without apathy (PDA−, MAES score ≤ 14) and with apathy (PDA+, MAES score > 14). Following MIRT training, the PDA– group demonstrated significantly improved motor function. The study confirmed that higher levels of apathy were correlated with less favorable rehabilitation outcomes, underscoring the detrimental effect of apathy on motor recovery in PWP. Further investigation into the mechanisms through which apathy impacts rehabilitation outcomes is essential for developing personalized rehabilitation strategies and improving overall treatment efficacy.
